# Genetic profile of motor neuron disease in a multiregional Brazilian cohort: an 18-year real-world experience in 1,911 patients

**DOI:** 10.3389/fneur.2026.1883977

**Published:** 2026-08-05

**Authors:** Christian Marques Couto, Elisa de Melo Queiroz, Maria Carolina da Cunha, Savana Camilla de Lima Santos, Alessandra De La Roque Ferreira, Osvaldo José Moreira Nascimento

**Affiliations:** 1 Neurology Department, SARAH Network of Rehabilitation Hospitals, Rio de Janeiro, Brazil; 2 Postgraduate Program in Neurology and Neurosciences, Faculty of Medicine, Fluminense Federal University (UFF), Rio de Janeiro, Brazil; 3 Translational Neuroscience Graduate Program (PGNET), Federal University of Rio de Janeiro (UFRJ), Rio de Janeiro, Brazil; 4 Molecular Biology Laboratory, SARAH Network of Rehabilitation Hospitals, Brasília, Brazil

**Keywords:** amyotrophic lateral sclerosis, ATXN2, Brazilian cohort, *C9orf72*, genetic testing, motor neuron disease, real-world evidence, SOD1

## Abstract

**Background:**

Genetic testing in amyotrophic lateral sclerosis (ALS) is increasingly recommended for all patients, but real-world implementation in middle-income countries remains heterogeneous. Brazilian motor neuron disease genetics has largely been characterized through protocol-based cohorts focused on the founder *VAPB* p.Pro56Ser variant (amyotrophic lateral sclerosis type 8, ALS8). The broader genetic landscape beyond ALS8 in Brazilian clinical populations has not been described in a large multiregional cohort. We aimed to characterize this profile.

**Methods:**

We retrospectively analyzed 1,911 patients with motor neuron disease followed at six SARAH Network rehabilitation hospitals across Brazil between 2007 and 2024. Genetic testing was clinically directed; ALS8 cases (*n* = 78) were characterized separately. We assessed characteristics of genetically tested versus untested patients, the temporal evolution of testing modalities, and the diagnostic yield by gene and by Byrne family history classification.

**Results:**

Of 1,833 patients without ALS8, 318 (17.3%) underwent genetic testing, with selection bias toward younger age, higher educational attainment, and positive family history of ALS or frontotemporal dementia (all *p* < 0.001). Testing expanded markedly after 2020, particularly *C9orf72* screening and next-generation sequencing panels. Panel/exome sequencing identified pathogenic or likely pathogenic variants in 13 of 66 patients (19.7%), consistent with international literature for mixed familial-sporadic cohorts. Across all modalities, 30 patients without ALS8 carried pathogenic or likely pathogenic variants in six genes: *SOD1* (*n* = 15), *C9orf72* (*n* = 11; 7.0% of 157 tested), and *VCP, FUS, TBK1*, and *SETX* (*n* = 1 each). *ATXN2* intermediate-length CAG repeats were identified in 15 of 230 patients tested (6.5%), replicating the 6.3% previously reported in a Brazilian multicenter sample. Yield by Byrne classification followed a marked gradient (Definite: 89.7%, Probable: 24.0%, Possible: 15.4%, Sporadic: 4.9%; *p* < 0.001). The *SOD1* spectrum included recurrent variants of likely Iberian/European origin.

**Conclusion:**

This 18-year cohort defines a real-world genetic profile of motor neuron disease beyond ALS8, with diagnostic yield comparable to the literature and a Byrne gradient supporting universal testing. Beyond family history, socioeconomic factors appeared to shape access to testing, reinforcing the importance of equitable genetic testing for diagnostic precision, family counseling, and access to gene-targeted therapy.

## 1 Introduction

Amyotrophic lateral sclerosis (ALS) is the prototypical adult-onset motor neuron disease, characterized by progressive degeneration of upper and lower motor neurons and a median survival of 3–5 years from symptom onset (1, 2). It is a genetically heterogeneous disorder, with pathogenic variants in over 40 genes implicated in disease pathogenesis and an estimated heritability of 40–60% across population studies (3). Although approximately 5–10% of patients have a documented family history of ALS or a related condition, such as frontotemporal dementia, in a first- or second-degree relative, pathogenic variants are also identified in a meaningful proportion of patients without such a history, owing to incomplete penetrance, age-dependent expressivity, and the limited reach of family history ascertainment in routine clinical settings (3). A recent series of studies indicates that the molecular yield of comprehensive genetic testing ranges from approximately 60–70% in familial cases to 10–15% in apparently sporadic cases (4, 5).

This shift in genetic understanding has been paralleled by a shift in therapeutics. The accelerated approval of tofersen for *SOD1*-ALS by the U.S. Food and Drug Administration in April 2023 marked the first gene-targeted therapy for any form of ALS (6, 7), with regulatory decisions in other jurisdictions following thereafter. Additional antisense oligonucleotide programs targeting other ALS subtypes have advanced clinically, including ulefnersen for *FUS*-ALS, although early-phase trials of agents targeting *C9orf72* (BIIB078) and *ATXN2* (BIIB105) have been discontinued after failing to demonstrate clinical benefit (8). Even beyond approved gene-targeted therapies, molecular characterization informs prognostic counseling, family planning, and clinical trial eligibility. In this context, international consensus guidelines published in 2023 recommend genetic testing for all patients with ALS, regardless of family history, with a minimum panel that includes *C9orf72*, *SOD1*, *FUS*, and *TARDBP* (5, 9). However, real-world implementation of these recommendations remains heterogeneous, particularly in middle-income countries where access to multigene panels and repeat-expansion testing depends on local laboratory infrastructure, reimbursement frameworks, and clinical referral practices.

Brazil, a country of over 200 million people, offers a particularly informative setting for this question. In autosomal-marker studies summarized by Souza and colleagues, the Brazilian population shows weighted mean ancestry contributions of approximately 68% European, 20% African, and 12% Native American, with substantial regional variation (10), reflecting a demographic history that is poorly represented in the genomic databases that underpin modern variant interpretation. Globally, individuals of European ancestry account for the substantial majority of participants in genome-wide and clinical sequencing studies, with direct consequences for variant classification, polygenic risk estimation, and the interpretability of genetic findings in non-European populations (11). Brazil exemplifies this gap acutely: recent whole-genome sequencing of admixed Brazilian cohorts has identified more than 8 million variants absent from major reference databases, including tens of thousands predicted to be deleterious (12), and the implementation of precision medicine in this setting faces specific structural challenges related to demographic complexity, regional disparities, and uneven access to sequencing technologies (13). The Brazilian ALS spectrum has been shaped by founder events linked to colonial-era European migration and by subsequent African and Native American admixture, producing a genetic landscape that cannot be assumed to mirror European or East Asian series.

Within this context, the genetic landscape of motor neuron disease in Brazil has been characterized through a series of important but methodologically heterogeneous studies. The *VAPB* p.Pro56Ser variant was first identified by Nishimura and colleagues in 2004 as the cause of ALS type 8 and clinically variable motor neuron disease in Brazilian kindreds (14); a subsequent haplotype study demonstrated a common founder shared by families of Portuguese-Brazilian and African-Brazilian ancestry, dated to approximately 23 generations ago and consistent with the Portuguese colonization of Brazil (15). This founder variant accounts for 30–44% of familial ALS in selected Brazilian institutional series (16, 17), a frequency unique to Brazilian familial ALS, reflecting the founder origin of this variant. Beyond ALS8, the Brazilian genetic profile has been progressively characterized through targeted studies: *C9orf72* hexanucleotide expansion frequencies of 11.8% in familial and 3.6% in apparently sporadic ALS have been reported in a southeastern Brazilian multicenter sample (18); *ATXN2* intermediate-length CAG repeats (≥26) have been documented in 6.3% of patients within a multicenter Brazilian sample, conferring an approximately 2.5-fold increased risk of ALS (19); a *SOD1* p.Val120Leu founder cluster has been described in the Acaraú River Valley of northeastern Brazil (20, 21); a recurrent *ANXA11* p.Asp40Tyr variant of likely founder origin has been identified in approximately 4% of a southeastern Brazilian sample (17). However, these studies were largely protocol-based, conducted in research-grade settings, or restricted to specific genes or geographic regions; the broader real-world implementation of genetic testing, including who is actually tested, when testing is performed, which assays are used, and what diagnostic yield is achieved, has not been systematically described in a large multiregional Brazilian cohort.

Against this background, we describe the genetic profile of motor neuron disease in an 18-year retrospective cohort followed at the SARAH Network of Rehabilitation Hospitals across multiple Brazilian regions. Given the historical and quantitative prominence of ALS8/*VAPB*-related familial ALS in our institutional series, this subgroup has been characterized separately and is reported elsewhere (22); the present study focuses on the broader genetic spectrum beyond ALS8. Specifically, we aimed to: (i) describe the demographic and clinical characteristics of patients who did and did not undergo genetic testing, including a descriptive sub-analysis of patients with documented parental consanguinity, and identify the factors associated with access to testing in this routine clinical setting; (ii) characterize the temporal evolution of genetic testing modalities over time, capturing the impact of regulatory and technological changes on routine clinical practice; (iii) examine the influence of family history classification, as proposed by Byrne and colleagues (23), on the empirical patterns of genetic testing decisions and on the diagnostic yield achieved across this cohort; (iv) report the frequency of pathogenic and likely pathogenic variants identified, organized by gene and by phenotype.

## 2 Methods

### 2.1 Study design and population

This retrospective observational study included patients with motor neuron disease (MND) evaluated at the SARAH Network of Rehabilitation Hospitals between January 2007 and December 2024, spanning 18 calendar years of clinical follow-up. Patients were evaluated at six specialized SARAH Network hospitals covering a registry of 1,911 patients drawn from 25 Brazilian states and the Federal District. We included all patients with a clinical diagnosis of ALS, primary lateral sclerosis (PLS), progressive muscular atrophy (PMA), flail arm syndrome, or flail leg syndrome who underwent at least one consultation with the institutional motor neuron disease team during the study period. Because patients were enrolled at the time of referral rather than at symptom onset, this represents a prevalent cohort design. The study was approved by the local ethics committee (CAAE 09814119.9.1001.0022). The requirement for informed consent was waived under Brazilian Resolution 466/12 because the study used retrospective deidentified data.

### 2.2 Clinical characterization

Demographic data, disease characteristics, and clinical phenotype were collected through standardized retrospective chart review. The clinical diagnosis of ALS followed the El Escorial revised criteria (24). Clinical phenotype was classified according to a modified version of the criteria proposed in our previous multicenter Brazilian cohort (25) into eight categories: spinal-onset ALS, bulbar-onset ALS, cognitive-onset ALS, respiratory-onset ALS, PLS, PMA, flail arm syndrome, and flail leg syndrome. The category of cognitive-onset ALS, defined as ALS in which cognitive or behavioral symptoms preceded or coincided with motor onset, overlaps with the broader ALS–frontotemporal spectrum disorder framework proposed by Strong et al. (26).

For *C9orf72* carriers, cognitive function was clinically assessed and classified according to DSM-5 criteria (27) as cognitively intact, mild neurocognitive disorder, or major neurocognitive disorder (dementia); when formal cognitive screening was available in the patient record, it was incorporated into the DSM-5 classification. Additionally, behavioral assessment was undertaken by retrospective chart review, conceptually framed by the criteria proposed by Strong et al. (26), and considered behavioral changes (apathy, disinhibition, loss of empathy, perseverative behavior, hyperorality, or personality changes) and psychotic features (delusions, hallucinations, or thought disorganization).

Family history of motor neuron disease or frontotemporal dementia (FTD) was classified according to the criteria into four categories—Definite, Probable, Possible familial, or Sporadic—based on the degree of kinship and the number of affected relatives, as proposed by Byrne et al. (23). Under these criteria, Definite familial disease corresponds to three or more affected first- or second-degree relatives, Probable to two affected relatives, and Possible to a single additional affected relative, while the Sporadic category corresponds to the absence of any first- or second-degree relative affected by ALS or FTD. Patients whose family history could not be reliably ascertained—in this cohort, three patients who were adopted—were not assigned to any Byrne category and were excluded from the family-history-stratified analyses. Unlike the original formulation, which also permits classification as familial in the presence of a known pathogenic variant even without other affected relatives, classification here was based exclusively on the pattern of familial aggregation, independent of genetic test results, in line with our previous cohort studies (22, 25). This approach preserves the independence between family history and molecular findings, a necessary condition for the non-circular calculation of diagnostic yield across Byrne strata. Parental consanguinity was self-reported by patients or family members during clinical evaluation and was defined as a situation in which the biological parents of the proband were related as second cousins or closer, corresponding to an expected coefficient of inbreeding in the offspring of *F* ≥ 1/64 (≈ 0.0156), in line with standard clinical genetics definitions (28, 29). Parental consanguinity was analyzed as an exploratory clinical variable because of its relevance to recessive neurogenetic disorders and to genetic-testing prioritization.

### 2.3 Genetic testing

Genetic testing was performed pragmatically and clinically directed throughout each patient’s clinical history, reflecting real-world practice rather than a predefined research protocol. Tests were performed primarily at the molecular genetics laboratory of the SARAH Network; tests obtained externally by patients at accredited clinical laboratories were reviewed by the investigators and accepted when they met established quality criteria. Some patients had been tested before referral to the SARAH Network, particularly through linkage analyses in informative pedigrees, thereby accounting for the testing window (2005–2025) extending beyond the admission window (2007–2024). Genetic test results were compiled and reviewed through February 2026, accounting for panels performed in late 2025, whose results became available only thereafter. Testing modalities included targeted single-gene Sanger sequencing for *VAPB* (p.Pro56Ser; ALS8), repeat-primed PCR for *C9orf72* hexanucleotide expansions, *ATXN2* CAG repeat sizing, multigene panels, and whole-exome sequencing. Multigene panels were performed at five accredited Brazilian laboratories, including the SARAH Network laboratory, and covered between 18 and 169 genes, depending on the laboratory and date of testing; the largest panels included additional neuromuscular disease genes beyond those associated with ALS. The most frequently included ALS-associated genes were *VAPB, SOD1, FUS, TARDBP, VCP, TBK1, SETX, ALS2, SPG11, SIGMAR1, OPTN, PFN1*, and *UBQLN2*; coverage of the population-relevant gene *ANXA11* varied across panels and laboratories. The composition, gene count, period of use, and laboratory of each panel are summarized in Supplementary Table S1.

For *ATXN2* CAG repeat sizing, the principal threshold for intermediate-length alleles was set at ≥26 CAG repeats, in alignment with the only previous Brazilian ALS study (19). Because thresholds for ALS-associated *ATXN2* intermediate alleles vary across the literature, three additional thresholds were prespecified for sensitivity analyses: ≥27 repeats, corresponding to the lower bound of the range originally proposed by Elden and colleagues; 29–33 repeats, examined in the meta-analysis by Sproviero and colleagues; ≥31 repeats, a stricter cutoff supported by recent large-scale genomic data. *ATXN2* intermediate alleles were analyzed as risk-factor findings and were not counted as pathogenic or likely pathogenic diagnostic variants (30–32). Alleles ≥34 CAG repeats, corresponding to the established pathogenic range for spinocerebellar ataxia type 2 (SCA2), were not observed in this cohort.

Variant interpretation followed the joint guidelines of the American College of Medical Genetics and Genomics and the Association for Molecular Pathology (ACMG–AMP) (33). For the present analysis, we considered variants that meet ACMG–AMP criteria for these classifications to be pathogenic or likely pathogenic, and we also included patients identified through linkage analysis in informative pedigrees (for *SOD1*) and patients clinically diagnosed within well-characterized informative pedigrees consistent with the founder *VAPB* p.Pro56Ser variant (for ALS8). Variants of uncertain significance were retained in supplementary analyses but were not included in the primary diagnostic yield calculations.

### 2.4 Statistical analysis and diagnostic yield calculation

Continuous variables are described as median and interquartile range (IQR); categorical variables as absolute and relative frequencies. Comparisons between genetically tested and untested patients used the Mann–Whitney U test for continuous variables and Pearson’s χ^2^ test or Fisher’s exact test for categorical variables, as appropriate. The diagnostic yield of comprehensive panel or whole-exome sequencing was calculated as the proportion of unique patients tested who carried a pathogenic or likely pathogenic variant; one patient who underwent both panel and exome sequencing was counted once in the deduplicated denominator. The diagnostic yield by family history classification was compared using the χ^2^ test for trend, with Wilson 95% confidence intervals calculated for the proportions. Statistical analyses were performed using JAMOVI (version 2.6.44) and Python (version 3.11) with the Pandas Library. Cumulative test counts reflect all assays performed, whereas analytical denominators in yield calculations refer to unique non-ALS8 patients with valid results for each assay. A *p*-value < 0.05 was considered statistically significant.

## 3 Results

### 3.1 Cohort characteristics and genetic testing pattern

The analyzed cohort comprised 1,911 patients followed at the SARAH Network between 2007 and 2024. Median age at symptom onset was 55 years (IQR: 47–63), with a slight male predominance (male: female ratio: 1.2:1). By clinical phenotype, 1,219 patients had spinal-onset ALS (63.8%), 262 bulbar-onset ALS (13.7%), 166 progressive muscular atrophy (8.7%), 98 primary lateral sclerosis (5.1%), 88 flail arm syndrome (4.6%), 53 flail leg syndrome (2.8%), 16 cognitive-onset ALS (0.8%), and nine respiratory-onset ALS (0.5%).

**Figure 1:**
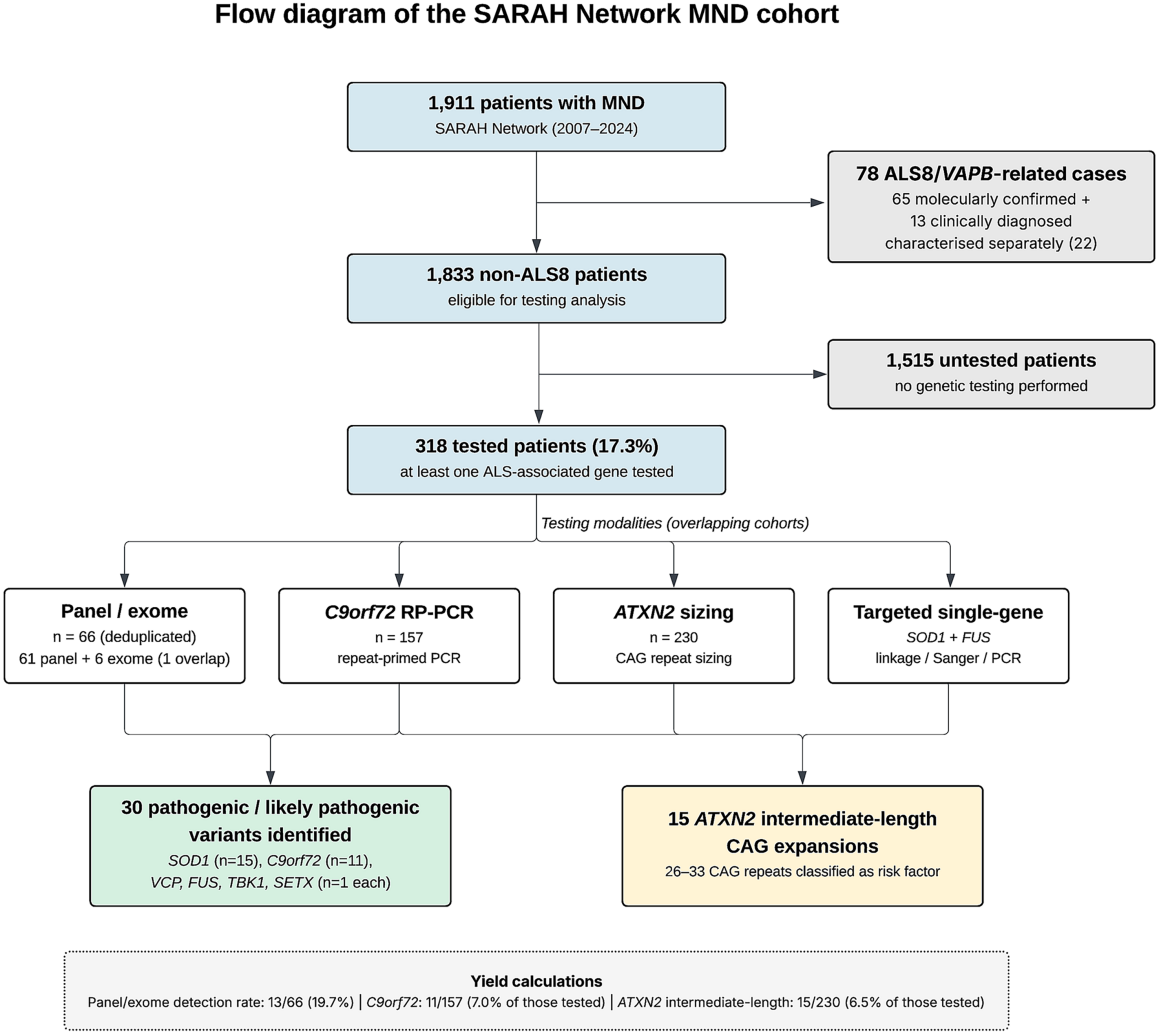
Flow diagram of the SARAH Network motor neuron disease cohort (admission window: 2007–2024, testing window: 2005–2025). Of 1,911 patients, 78 ALS8/*VAPB*-related cases (65 molecularly confirmed; 13 clinically diagnosed) were characterized separately (22) and excluded, leaving 1,833 patients without ALS8, of whom 318 (17.3%) underwent at least one genetic test. Testing modalities (panel/exome, *C9orf72 RP-PCR, ATXN2* sizing, targeted single-gene) shared cohorts; the panel/exome denominator (*n* = 66) has been deduplicated. Six pathogenic findings identified through targeted single-gene assays (4 *SOD1* by linkage analysis, 1 *SOD1* by Sanger, 1 *FUS* by targeted PCR) are included in the *n* = 30 total but excluded from formal yield calculations. *ATXN2* intermediate-length CAG repeats are reported separately as a risk factor. RP-PCR, repeat-primed polymerase chain reaction.

Genetic testing was performed in 383 patients (20.0% of the cohort), of whom 65 carried the *VAPB* p.Pro56Ser variant (ALS8), confirmed by molecular testing; an additional 13 ALS8 patients were identified clinically based on phenotype, family history, and pedigree without formal molecular confirmation, for a total of 78 ALS8 patients. Given that ALS8 is the most prevalent inherited form of ALS in this cohort and is the subject of a separate companion analysis (22), all 78 patients with ALS8 were excluded from the comparative analysis of genetically tested versus untested subgroups. After this exclusion, 318 of 1,833 patients without ALS8 (17.3%) had been tested for at least one ALS-associated gene (Figure 1). Tested patients differed significantly from untested patients across several dimensions (Table 1). They were younger at symptom onset (median: 52 vs. 57 years; *p* < 0.001), had a longer diagnostic delay (median: 17.1 vs. 15.5 months; *p* = 0.015), and were more frequently classified as familial cases by the Byrne criteria (combined Definite, Probable, or Possible: 16.4% vs. 7.7%; *p* < 0.001). Phenotypic distribution also differed (*p* < 0.001), with primary lateral sclerosis substantially over-represented among tested patients (14.2% vs. 3.5%) and progressive muscular atrophy under-represented (4.7% vs. 7.9%). Regarding educational attainment, the median number of years of schooling was identical between groups (11 years), but the distribution differed markedly (mean 10.6 vs. 9.26 years; *p* < 0.001 by Mann–Whitney U test), with 28.6% of untested patients having either no formal schooling (illiterate) or only incomplete primary education (1–4 years), compared with 15.4% in the tested group (χ^2^ test, *p* < 0.001). Sex distribution and consanguinity (parents related as second cousins or closer; *F* ≥ 1/64) did not differ significantly between groups.

**Table 1:** Demographic and clinical characteristics of genetically tested versus untested patients (excluding ALS8/*VAPB*-related patients; *n* = 1,833).

Variable	Untested (*n* = 1,515)	Tested (*n* = 318)	*p*-value
Demographics			
Sex, male, *n* (%)	832 (54.9)	162 (50.9)	0.218
Age at symptom onset, years, median (IQR)	57 (47–64)	52 (43–61)	<0.001
Disease characteristics			
Diagnostic delay, months, median (IQR)	15.5 (9.6–26.3)	17.1 (10.0–32.9)	0.015
Clinical phenotype, *n* (%)			<0.001
Spinal-onset ALS	992 (65.5)	185 (58.2)	
Bulbar-onset ALS	221 (14.6)	41 (12.9)	
Progressive muscular atrophy (PMA)	120 (7.9)	15 (4.7)	
Primary lateral sclerosis (PLS)	53 (3.5)	45 (14.2)	
Flail arm syndrome	72 (4.8)	16 (5.0)	
Flail leg syndrome	38 (2.5)	10 (3.1)	
Cognitive-onset ALS	11 (0.7)	5 (1.6)	
Respiratory-onset ALS	8 (0.5)	1 (0.3)	
Family history			
Byrne classification, *n* (%)			<0.001
Definite	49 (3.2)	15 (4.7)	
Probable	48 (3.2)	24 (7.5)	
Possible	19 (1.3)	13 (4.1)	
Sporadic	1,399 (92.3)	266 (83.6)	
Consanguinity			
Present (F ≥ 1/64), *n* (%)	31 (2.0)	7 (2.2)	0.831
Educational attainment			
Education level, *n* (%)			<0.001
Illiterate or incomplete primary (0–4 years)	433 (28.6)	49 (15.4)	
Complete primary (5–8 years)	219 (14.5)	40 (12.6)	
Secondary (9–11 years)	477 (31.5)	124 (39.0)	
Higher (≥12 years)	386 (25.5)	105 (33.0)	

Continuous variables were compared by the Mann–Whitney U test; categorical variables were compared by Pearson’s *χ*^2^ test or Fisher’s exact test, as appropriate. ALS, amyotrophic lateral sclerosis; ALS8, amyotrophic lateral sclerosis type 8; F, coefficient of inbreeding; FTD, frontotemporal dementia; IQR, interquartile range; PLS, primary lateral sclerosis; PMA, progressive muscular atrophy.

**Figure 2:**
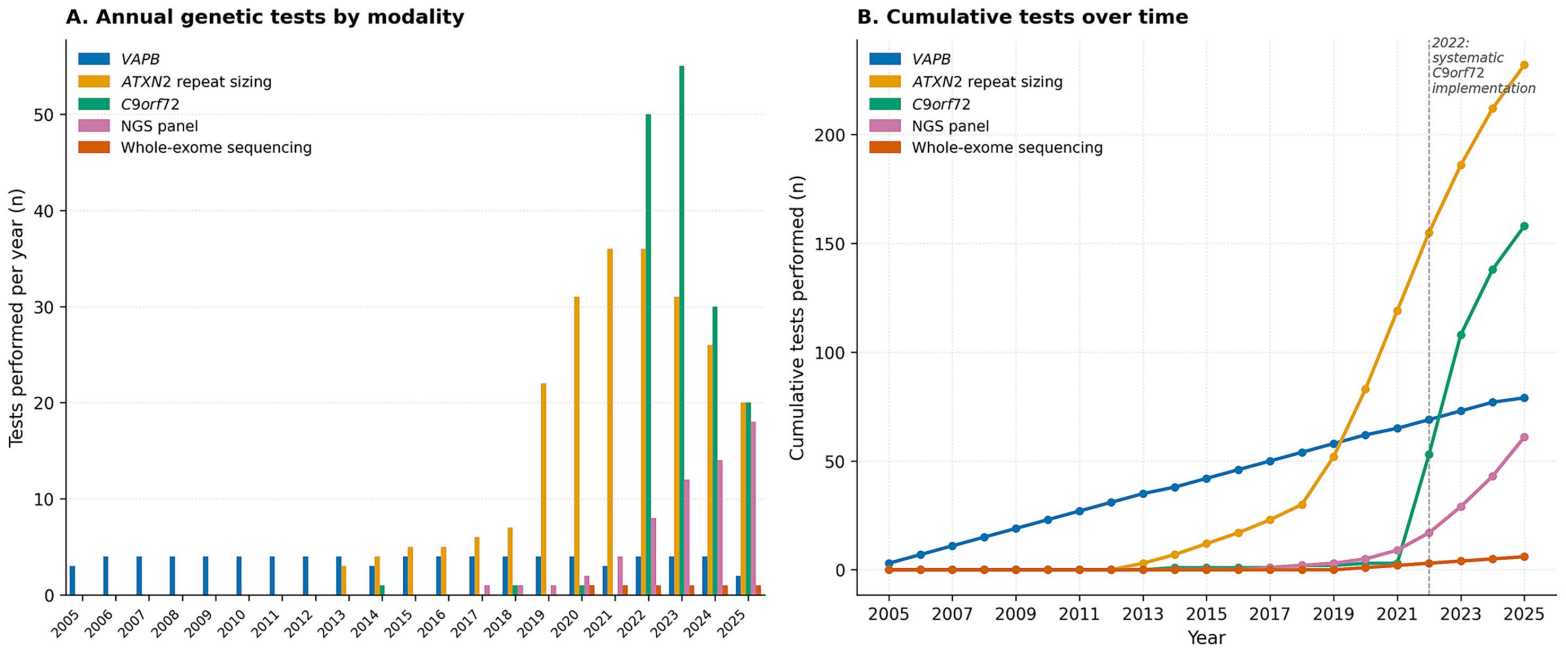
Temporal evolution of genetic testing in the SARAH Network motor neuron disease cohort (2005–2025). **(A)** Annual number of tests performed, by modality. **(B)** Cumulative tests over time. The vertical dashed line marks the year (2022), when systematic *C9orf72* screening was implemented at our institution. *VAPB* testing began in 2005 and *ATXN2* sizing in 2012; multigene NGS panels and *C9orf72* RP-PCR became routinely available after 2020 and 2022, respectively. NGS, next-generation sequencing; RP-PCR, repeat-primed polymerase chain reaction.

The modalities of genetic testing evolved markedly over the study period (Figure 2). Single-gene Sanger sequencing for *VAPB* p.Pro56Ser was the first modality available to patients in our cohort, with cumulative testing reaching 79 patients by 2025. Repeat-primed PCR for *C9orf72* hexanucleotide expansions remained largely unavailable until 2014; from 2022 onwards, the cumulative number of tests rose markedly to 157 by 2025. Next-generation sequencing (NGS) panels remained virtually absent until 2020 and were concentrated in 2022–2025 (61 cumulative tests). Whole-exome sequencing remained a rare resource, with only six tests performed throughout the entire period; one patient underwent both panel and exome sequencing, yielding a deduplicated denominator of 66 unique patients with comprehensive panel or exome testing.

### 3.2 Diagnostic yield by family history classification

**Figure 3:**
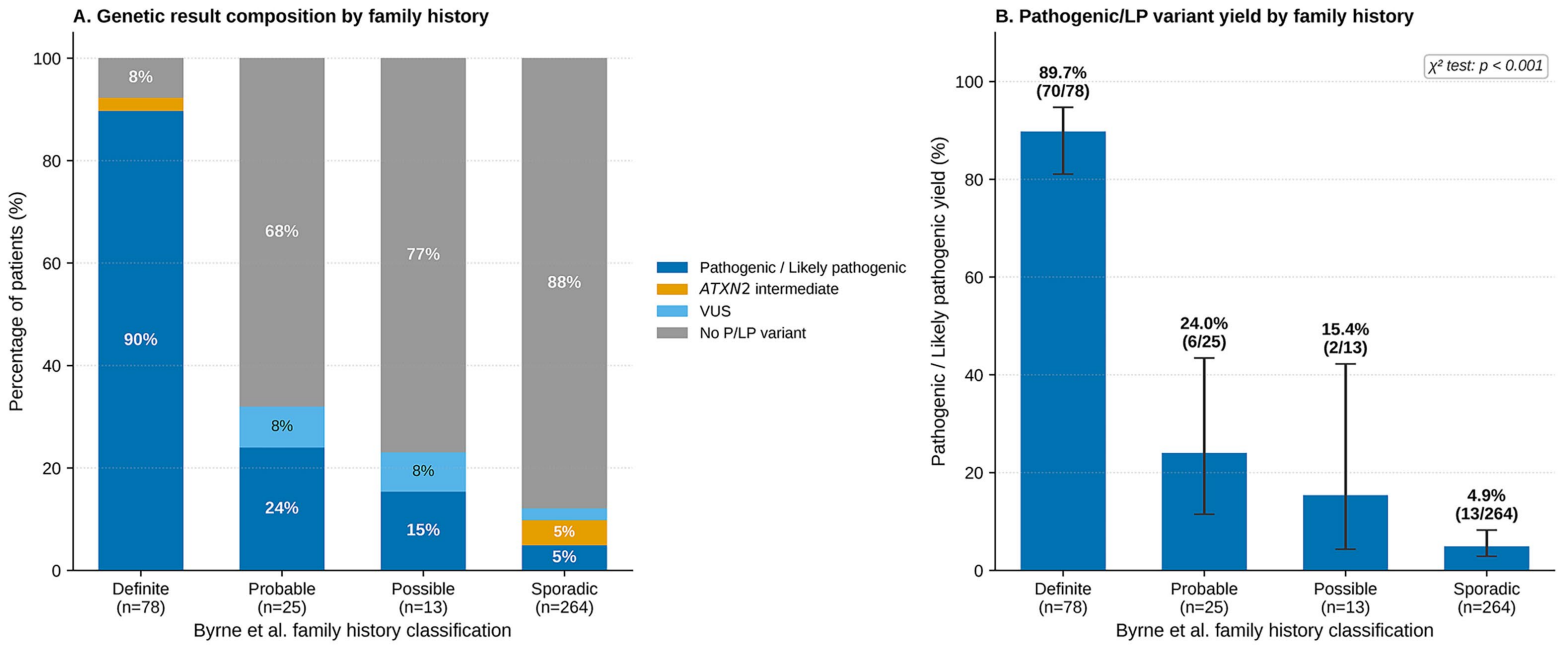
Genetic diagnostic yield according to family history classification (Byrne criteria) in the tested subcohort (*n* = 380). **(A)** Composition of genetic results by Byrne category. *ATXN2* intermediate-length alleles, classified separately as a risk factor, were predominantly identified among Sporadic cases. **(B)** Pathogenic/likely pathogenic variant yield by Byrne category, with 95% Wilson confidence intervals. χ^2^ test, *p* < 0.001.

Among the 380 patients tested who could be classified by family history (3 of the 383 tested were excluded, as detailed in the Methods section), the diagnostic yields of pathogenic or likely pathogenic variants varied substantially according to family history classification (Figure 3). For this family-history-stratified analysis, patients with ALS8 were retained within their corresponding Byrne categories, since family history is intrinsic to the Byrne classification, and excluding the predominantly familial *VAPB* cases would distort the relationship between familial evidence and molecular findings. In patients with Definite familial ALS by the Byrne criteria (23), 70 of 78 (89.7%) carried a pathogenic or likely pathogenic variant, predominantly the *VAPB* p.Pro56Ser variant. Three patients with non-ascertainable family history, all due to adoption, were excluded from this classification. This rate decreased progressively across categories of weaker familial evidence: 24.0% (6/25) in Probable familial cases, 15.4% (2/13) in Possible familial cases, and 4.9% (13/264) in Sporadic cases (i.e., without documented family history). *ATXN2* intermediate-length alleles were identified predominantly in patients without family history (13/15 carriers; 86.7%). The differences in result composition across these categories were highly significant (χ^2^ test, *p* < 0.001). The same gradient persisted when patients with ALS8 were excluded from the analysis (Definite: 7/15 [46.7%], Probable: 5/24 [20.8%], Possible: 2/13 [15.4%], Sporadic: 12/263 [4.6%]), confirming that the association between familial evidence and diagnostic yield is not driven solely by the founder VAPB variant.

### 3.3 Genetic findings

Considering the genetically tested cohort as a whole (*n* = 383, including ALS8), pathogenic or likely pathogenic variants were identified in 108 patients (28.2% of those tested; 5.7% of the full cohort of 1,911 patients). ALS8/*VAPB* accounted for the majority (78 patients; 20.4% of those tested, 4.1% of the cohort), followed by *SOD1* (15; 3.9 and 0.8%) and *C9orf72* (11; 2.9 and 0.6%). *ATXN2* intermediate-length expansions, considered a risk factor rather than a pathogenic variant, were present in 15 patients (3.9% of those tested, 0.8% of the cohort). The clinical phenotype, survival, and natural history of the ALS8 subgroup have been characterized in a dedicated companion study (22); here, ALS8 cases were quantified within the overall genetic burden but were not the focus of the comparative testing analyses below. These cohort-level proportions are summarized in Supplementary Figure S1.

Pathogenic or likely pathogenic variants were identified in 30 patients without ALS8 across six genes (*SOD1*, *VCP*, *FUS*, *TBK1*, *SETX*, and *C9orf72*); an additional 15 patients carried *ATXN2* intermediate-length CAG repeat expansions (classified as a risk factor rather than a pathogenic variant). Among patients without ALS8 who underwent comprehensive panel or whole-exome sequencing, the diagnostic yield was 13/66 (19.7%). Pathogenic or likely pathogenic variants identified in this cohort are summarized by gene in Table 2. In addition, 10 variants of uncertain significance (VUS) were identified in six ALS-associated genes (*ALS2*, *OPTN*, *PFN1*, *SETX*, *SOD1*, and *TBK1*) and are listed in Supplementary Table S2; one patient harbored both a *SOD1* likely pathogenic variant and an *ALS2* VUS.

**Table 2:** Genetic findings in non-ALS8 patients of the SARAH Network motor neuron disease cohort.

Gene	Variant (HGVS)	*n* patients (*n* families)	ACMG/classification	Predominant phenotype (onset age range, years)
*SOD1*	p.(Phe21Cys)	2 (2)	P	Lumbar-onset ALS (32–43)
*SOD1*	p.(Leu38Val)	1 (1)	P	Lumbar-onset ALS (37)
*SOD1*	p.(Asp91Ala)^*^	1 (1)	LP	Lumbar-onset ALS (55)
*SOD1*	p.(Asp91Val)	1 (1)	P	Lumbar-onset ALS (50)
*SOD1*	p.(Gly94Cys)	2 (1)	P	Progressive muscular atrophy (52–60)
*SOD1*	p.(Glu101Lys)	1 (1)	P	Cervical-onset ALS (42)
*SOD1*	p.(Leu107_Ser108del)	1 (1)	P	Cervical-onset ALS (45)
*SOD1*	p.(Val120Leu)	2 (2)	LP	Lumbar-onset ALS (37–60)
*SOD1*	p.(Glu133del)	1 (1)	P	Lumbar-onset ALS (44)
*SOD1*	p.(Thr138Ile)	1 (1)	LP	Progressive muscular atrophy (50)
*SOD1*	p.(Ile150Thr)	2 (1)	LP	Cervical-onset ALS (32–41)
*VCP*	p.(Arg155Cys)	1 (1)	P	Lumbar-onset ALS (39)
*FUS*	p.(Arg521His)	1 (1)	P	Cervical-onset ALS (47)
*TBK1*	p.(Val421CysfsTer27)	1 (1)	LP	Bulbar-onset ALS (38)
*SETX*	c.5274_5274 + 1del (splice-site)	1 (1)	LP	Lumbar-onset ALS (43)
*C9orf72*	GGGGCC expansion	11 (11)	P^†^	Spinal-onset ALS (5), bulbar-onset ALS (2), cognitive-onset ALS (2), PLS (1), flail leg (1) (43–71)
*ATXN2*	(CAG)31–33	7 (6)	RF^‡^	Spinal-onset ALS (38–73)
*ATXN2*	(CAG)27–30	5 (5)	RF^‡^	Spinal-onset ALS, flail arm (43–63)
*ATXN2*	(CAG)26	3 (3)	RF^‡^	Spinal-onset ALS, flail leg (52–65)

^*^c.272A>C (SOD1 NM_000454.5; ClinVar VCV000014766), historically also reported as p.Asp90Ala (D90A). ^†^Recognized pathogenic hexanucleotide repeat expansion; not formally classifiable under ACMG–AMP guidelines, which were developed for sequence variants. ^‡^Risk factor; not classifiable under ACMG–AMP guidelines. ACMG, American College of Medical Genetics and Genomics; ALS, amyotrophic lateral sclerosis; ALS8, amyotrophic lateral sclerosis type 8; AMP, Association for Molecular Pathology; HGVS, Human Genome Variation Society; LP, likely pathogenic; P, pathogenic; PLS, primary lateral sclerosis; PMA, progressive muscular atrophy; RF, risk factor.

#### 3.3.1 SOD1

Pathogenic or likely pathogenic variants in *SOD1* were identified in 15 patients from 13 unrelated families. Of these, 10 patients were detected through multigene panel or exome sequencing, corresponding to a panel/exome detection rate of 15.2% (10/66); four additional patients were identified through linkage analysis in informative pedigrees, and one patient was identified by single-gene Sanger sequencing. Eleven distinct variants were detected, distributed across exons 1, 2, 4, and 5; all patients were heterozygous.

**Figure 4:**
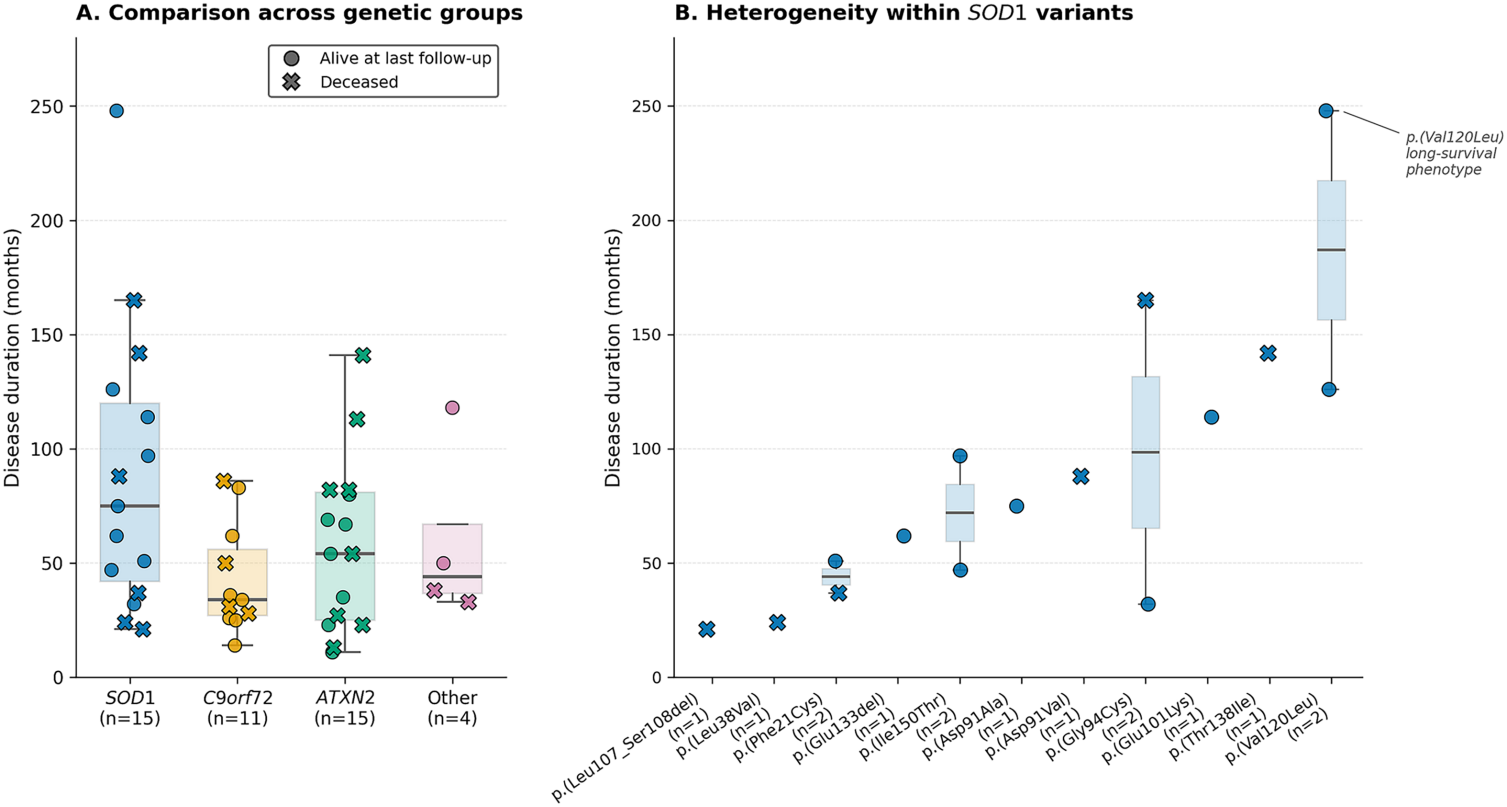
Disease duration distribution by genetic finding in 45 patients with pathogenic, likely pathogenic, or intermediate-risk variants. **(A)** Disease duration across four genetic groups: *SOD1* (*n* = 15), *C9orf72* (*n* = 11), *ATXN2* intermediate alleles (*n* = 15), and other genes combined (*VCP, FUS, TBK1, SETX*; *n* = 4). **(B)** Heterogeneity within *SOD1*: disease duration by individual variant, ordered by ascending median. Box plots show median, interquartile range, and whiskers (1.5 × IQR); individual patients are overlaid as filled circles (alive at last follow-up) or crosses (deceased). Disease duration is defined as the months from symptom onset to the last follow-up or death. The long-survival phenotype associated with *SOD1* p.(Val120Leu) is annotated. Statistical comparison between groups was not performed due to small group sizes. IQR, interquartile range.

Across all *SOD1* carriers, the median age at symptom onset was 44 years (IQR: 39–51), substantially younger than the cohort-wide median of 55 years; female predominance was marked (10/15; 66.7%). Lower limb onset was the dominant presentation (11/15), and three patients had a phenotype of progressive muscular atrophy. Disease duration was heterogeneous (range 21–248 months) (Figure 4); six patients had died at the time of analysis.

Two pairs of unrelated families harbored the same variant: p.(Phe21Cys) and p.(Val120Leu); both are recurrent variants of likely Iberian/European origin in our cohort, with p.(Val120Leu) consistent with the previously reported Acaraú River Valley founder cluster in northeastern Brazil (20, 21). Two additional families included more than one genetically confirmed affected relative: a father–son pair carrying p.(Gly94Cys) with progressive muscular atrophy in both, and two relatives carrying p.(Ile150Thr). The patient harboring p.(Thr138Ile) presented at 50 years of age with lumbar onset and a slowly progressive ascending phenotype meeting criteria for progressive muscular atrophy, with late bulbar and respiratory involvement, classification as definite familial ALS by Byrne criteria, and a prolonged disease course of 143 months until death.

Both p.(Val120Leu) carriers presented with lower-limb-onset and slowly progressive disease courses (disease durations of 248 and 126 months at last assessment); one was born in Galicia, Spain, of Spanish parents. Two further patients carried distinct missense variants affecting codon 91: heterozygous p.(Asp91Ala) (historically also known as p.Asp90Ala or D90A) and heterozygous p.(Asp91Val), corresponding to two distinct substitutions of the same aspartate residue. The p.(Asp91Ala) carrier was reclassified during cohort review from VUS to likely pathogenic following ACMG–AMP re-evaluation (34–36); she presented at 55 years with classical lower-limb-onset ALS and was alive 75 months after symptom onset. The p.(Asp91Val) carrier presented at 50 years with lumbar-onset ALS and died after 88 months of disease.

#### 3.3.2 Other ALS-associated genes

Pathogenic or likely pathogenic variants in *VCP*, *FUS*, *TBK1*, and *SETX* were identified in four patients. The *FUS* variant was detected by targeted single-gene PCR, while the variants in the three remaining genes were detected by multigene panel or exome sequencing [panel/exome detection rate: 4.5% (3/66)]. All four patients were men with an onset between 38 and 47 years of age, substantially younger than the cohort-wide median.

One patient carried *VCP* p.(Arg155Cys), presenting with isolated lumbar-onset ALS without clinical evidence of myopathy or Paget’s disease; serum creatine kinase was mildly elevated (490 U/L). A second patient carried *FUS* p.(Arg521His) and was the only patient in this subgroup with a positive family history (Definite); this patient had rapid disease progression and died 38 months after symptom onset. A third patient carried a frameshift variant in *TBK1* (p.[Val421CysfsTer27]), with bulbar-onset ALS at 38 years and behavioral changes during follow-up in the absence of formally documented cognitive impairment. The fourth patient carried a splice-site variant in *SETX* (c.5274_5274+1del), with adult-onset ALS at 43 years, no family history, and was alive 50 months after symptom onset.

#### 3.3.3 C9orf72

The *C9orf72* hexanucleotide repeat expansion was identified in 11 of 157 patients tested by repeat-primed PCR (7.0% of those tested). Of the carriers, seven (63.6%) had a positive family history meeting the Byrne criteria for definite, probable, or possible familial cases (Definite: 3, Probable: 3, Possible: 1); the remaining four (36.4%) were classified as Sporadic by the same criteria.

Among the 11 carriers, one presented with a phenotype of primary lateral sclerosis, characterized separately in our Brazilian PLS cohort (37), and two presented with cognitive-onset ALS, both later meeting full criteria for ALS-FTD (26).

Cognitive function was assessed in all 11 carriers. Major neurocognitive disorder (dementia) was documented in three patients (27.3%; including the two ALS-FTD patients above and one additional patient), mild neurocognitive disorder (MCI) in two patients (18.2%), and the remaining six patients (54.5%) were cognitively intact. Behavioral impairment was documented in 4 of 11 carriers (36.4%) and partially overlapped with the cognitive impairment subgroup.

#### 3.3.4 *ATXN2* intermediate-length expansions

*ATXN2* intermediate-length CAG repeat expansions were identified in 15 of 230 patients tested (6.5% of those tested). Repeat lengths ranged from 26 to 33 CAG units across the 15 carriers. Sensitivity analyses using more restrictive thresholds yielded carrier frequencies of 5.2% (≥27 CAG), 4.8% (29–33 CAG), and 3.0% (≥31 CAG); the principal ATXN2 carrier-frequency finding was retained across all thresholds (Supplementary Table S3).

The clinical phenotype was strikingly homogeneous. All 15 carriers presented with classical motor neuron disease, predominantly spinal-onset ALS (13 of 15), with one flail-arm and one flail-leg case. Median age at symptom onset was 56 years (IQR 45–62), comparable to the cohort-wide median. No patient exhibited cerebellar ataxia, extrapyramidal signs, or abnormal extraocular movements during follow-up; neuroimaging (magnetic resonance imaging, *n* = 14; computed tomography, *n* = 1) showed no cerebellar atrophy in any patient.

Disease duration in *ATXN2* carriers ranged from 11 to 141 months across all carriers (median 67 months in the ≥31 CAG group).

Family history was infrequent: only two carriers—a single sibling pair—were classified as Definite familial ALS.

### 3.4 Consanguinity in the cohort

Parental consanguinity was documented in 38 patients (2.0% of the cohort), of whom 33 (86.8%) had no family history of MND. Compared with non-consanguineous patients, consanguineous patients differed in two respects: a higher proportion in the 25–39-year onset category (26.3% vs. 12.8%; *p* = 0.023) and a higher proportion of “Possible” familial cases by Byrne classification (10.5% vs. 1.5%; *p* = 0.005) were observed; sex, median age at onset, phenotype, and region of birth did not differ. Only 7 of the 38 consanguineous patients (18.4%) underwent genetic testing, of whom just two underwent multigene panel testing; no pathogenic or likely pathogenic variant was identified. Additional demographic detail is provided in Supplementary Table S4.

## 4 Discussion

This 18-year multiregional cohort of 1,911 patients describes one of the largest Brazilian clinical series of motor neuron disease reported to date. Five principal findings emerge from this study. First, only 17.3% of patients without ALS8 underwent genetic testing during the study period, with marked selection bias toward younger age, higher educational attainment, positive family history, and primary lateral sclerosis phenotype. Second, as an emergent observation in this real-world cohort, the modalities of genetic testing evolved markedly over the study period, with a clear inflection in C9orf72 testing volume in 2022 and NGS panels becoming concentrated after 2020. Third, the diagnostic yield in patients undergoing comprehensive panel or exome testing was 19.7% (13 of 66), a value consistent with the international literature for mixed familial-sporadic clinical cohorts and reflecting selective, clinically driven testing in routine practice. Fourth, the diagnostic yield of the Byrne family history classification showed a sharp monotonic gradient from Definite familial to apparently sporadic cases (Figure 3), with approximately one in 20 patients lacking documented family history nonetheless harboring a pathogenic or likely pathogenic variant. Fifth, our *ATXN2* intermediate-length (≥26 CAG) frequency of 6.5% provides an independent Brazilian replication of the 6.3% reported by Tavares de Andrade et al. (19) in a multicenter Brazilian sample.

The expansion of genetic testing in this cohort followed three converging forces: greater availability of NGS and repeat-primed PCR in Brazilian laboratories, the growing local literature (17–19), and the 2023 international guidelines recommending universal testing (5, 9). Structural barriers, including cost, public-system reimbursement, and uneven laboratory access, still constrain equitable implementation.

The diagnostic yield gradient by family history classification has direct clinical implications. The rates observed across categories, high in Definite familial cases and low in Sporadic cases, are both consistent with the international literature (38). However, even in patients without a recognized family history of MND, this rate translates to roughly 5% carrying a pathogenic or likely pathogenic variant on testing, a clinically relevant proportion that supports universal testing recommendations. In this large multiregional Brazilian cohort, family history remained the dominant criterion driving clinical referral to genetic testing, suggesting a pattern that may systematically underdiagnose patients who would benefit from molecular characterization, including those potentially eligible for gene-targeted therapy. Beyond family history, educational attainment also differed significantly between groups (mean 10.6 vs. 9.26 years; *p* < 0.001), with low-schooling patients representing 28.6% of the untested group versus 15.4% of the tested. This may reflect greater patient awareness and more active pursuit of testing among more educated individuals, combined with disparate access to private laboratories when public-system coverage is limited, pointing to socioeconomic, not only clinical, determinants of testing equity. Disparities in access to neurogenetic testing have been previously documented in high-income healthcare settings (39); in Brazilian admixed populations served by a heterogeneous mix of public and private systems, additional dimensions of inequity may emerge that warrant systematic characterization in future studies.

The *SOD1* variant spectrum identified here is notable for the recurrence of variants previously reported in Iberian or southern European contexts. Both p.(Val120Leu) carriers had lower-limb-onset, slowly progressive, lower-motor-neuron-predominant disease, with disease durations of 126 and 248 months and survival at last follow-up, mirroring indolent phenotypes described in Spanish and Brazilian reports of this variant (20, 21, 34). Notably, in one family [p.(Val120Leu)], the proband was born in Galicia, Spain, providing an internal link between this cohort and previously reported Iberian cases, and distinguishing this family from the Acaraú River Valley founder cluster described in northeastern Brazil (20, 21). Codon 91 was also represented by two distinct substitutions, p.(Asp91Ala) and p.(Asp91Val), in unrelated patients. The heterozygous p.(Asp91Ala) carrier was reclassified during cohort review from VUS to likely pathogenic following *SOD1*-specific ACMG–AMP re-evaluation (34), supported by reports of heterozygous D91A carriers presenting with ALS in non-Scandinavian European and Mediterranean populations (35, 36). Taken together with reports of these variants in southern European cohorts, our findings are compatible with, although do not prove, the hypothesis that part of the Brazilian *SOD1* spectrum reflects Iberian/European founder contributions within an admixed population (10). European ancestry contributions in the Brazilian Northeast have been characterized as predominantly Iberian in origin (40), an inference that warrants verification by genome-wide haplotypic analysis in future studies.

A further relevant observation was the p.(Thr138Ile) carrier, who presented with a slowly progressive lower-motor-neuron-predominant phenotype consistent with definite familial ALS by Byrne criteria. Although p.(Thr138Ile) is listed in clinical variant databases and regulatory datasets of *SOD1*-ALS, we did not identify a detailed indexed case description focused on this substitution; our observation, therefore, adds clinical detail to the phenotypic characterization of this variant, while remaining preliminary. Figure 4 illustrates substantial heterogeneity of disease duration both across genetic groups and within *SOD1* variants, reinforcing the practical relevance of variant-specific prognostic information for genetic counseling and for timely consideration of *SOD1*-targeted therapy (7).

Pathogenic or likely pathogenic variants in *VCP, FUS, TBK1*, and *SETX* were each identified in single patients, illustrating the long tail of rare causative genes contributing to the Brazilian ALS genetic spectrum. The *VCP* p.(Arg155Cys) variant identified here is the most extensively documented variant in the gene and with Paget’s disease of bone and frontotemporal dementia; the isolated ALS phenotype observed in our carrier, without myopathy or Paget’s disease, although with mild creatine kinase elevation that may reflect either active denervation or a subclinical myopathic component, underscores the well-recognized phenotypic heterogeneity of *VCP*-related disease. The *FUS* p.(Arg521His) variant in the principal mutational hotspot of the gene was associated with the rapidly progressive, family-history-positive ALS phenotype expected for this cluster of variants. Behavioral manifestations are recognized as part of the ALS–FTD spectrum associated with *TBK1* mutations and may occur in the absence of measurable cognitive decline (41, 42); this is consistent with the *TBK1* carrier in our cohort, who showed behavioral changes without formally documented cognitive impairment. Finally, the *SETX* splice-site variant in an adult-onset case without family history led us to favor the broader designation *SETX*-related ALS over the more restrictive ALS4 nomenclature, in line with the increasing recognition that *SETX* variants may produce phenotypes distinct from the classical juvenile-onset autosomal dominant form.

The *C9orf72* expansion frequency of 7.0% among tested patients in our cohort is intermediate between the 11.8% familial and 3.6% sporadic frequencies reported by Cintra et al. for pure ALS/MND in a multicenter Brazilian sample, consistent with the mixed family-history composition of our tested subgroup (18). *C9orf72* carriers were enriched for positive family history compared with the broader tested non-ALS8 cohort (7/11 [63.6%] vs. 52/318 [16.4%]), in keeping with the usual autosomal-dominant inheritance pattern of *C9orf72*-related disease and its recognized incomplete penetrance (43). However, 4 of 11 carriers (36.4%) had no documented family history that met the Byrne criteria, reinforcing the limitation of family history as a sole referral criterion for genetic testing. This is particularly relevant for *C9orf72*, in which apparently sporadic presentations may reflect incomplete penetrance, limited family-history ascertainment, or unrecognized FTD-spectrum disease in relatives. Of additional clinical interest, one carrier presented with a primary lateral sclerosis phenotype; this patient was characterized separately in our Brazilian PLS cohort and adds to the small number of reported *C9orf72*-positive cases presenting with PLS or pure upper-motor-neuron-predominant disease, including prior Brazilian observations (18, 37).

The agreement of our 6.5% finding with the frequency reported by Tavares de Andrade et al. provides observational support for the reproducibility of this *ATXN2* risk-factor signal in Brazilian ALS cohorts (19). The clinical phenotype of *ATXN2* carriers was strikingly homogeneous, with no signs suggestive of SCA2 in any patient, including among carriers with 32–33 repeats, supporting a practical clinical distinction between ALS-associated intermediate-length alleles and classical full-range expansions, and reinforcing the interpretation of intermediate alleles as risk modifiers rather than Mendelian diagnostic variants (30–32). *ATXN2* carriers descriptively showed longer disease duration than the *SOD1* and *C9orf72* groups (Figure 4); however, this observation should be interpreted with caution, as published data on survival in *ATXN2* intermediate-repeat carriers remain inconsistent. Chiò and colleagues reported shorter survival in Italian ALS patients carrying ≥31 polyQ repeats (44), whereas Glass and colleagues, in a large-scale genomic study, did not confirm earlier onset or shorter survival despite confirming the risk-factor association (32). Subgroup sizes, retrospective follow-up, and the absence of formal survival analysis preclude inference of a survival effect from these descriptive data.

An additional methodological consideration concerns the heterogeneous coverage of ALS-associated genes by the multigene panels effectively used in this cohort (Supplementary Table S1). The 61 patients tested by panel received assays from five accredited Brazilian laboratories (including the SARAH Network laboratory), and only two of these included *ANXA11*, a gene reported in approximately 4% of Brazilian familial ALS cases by Nunes Gonçalves et al. (17), with a recurrent variant of likely founder origin. This uneven coverage of population-relevant genes in our sample may have contributed to the underdiagnosis of *ANXA11*-related ALS and underscores the broader argument that the standardization of a minimum ALS gene set (*C9orf72, SOD1, FUS, TARDBP*), complemented by population-relevant genes such as *ANXA11* in panels offered to Brazilian patients, would represent a substantial advance for patient care.

Parental consanguinity was documented in 38 patients (2.0%) and was analyzed as an exploratory descriptive subgroup (Results 3.4; Supplementary Table S4). Compared with non-consanguineous patients, two differences reached statistical significance: a higher proportion of younger adult-onset and of “Possible” familial cases by Byrne classification; however, only one consanguineous patient had onset before age of 25, arguing against a strong signal of classical juvenile recessive ALS. These patterns suggest that consanguineous patients with younger adult onset or lower-motor-neuron-predominant phenotypes warrant consideration of motor neuron disease differential diagnoses, including hereditary motor neuropathies, adult-onset spinal muscular atrophy, and other recessive disorders that mimic ALS (45), through targeted assays beyond standard panels. These observations should be interpreted within the context of typical adult-onset ALS, in which parental consanguinity has lower standalone predictive value, since the most clinically relevant adult-onset forms are autosomal dominant with frequently reduced penetrance (46). Because the testing strategy applied to this subgroup focused predominantly on autosomal-dominant or risk-associated genes rather than systematic interrogation of recessive ALS-related genes such as *ALS2, SPG11*, or *SIGMAR1* (47), the absence of a pathogenic variant reflects limited ascertainment rather than evidence against recessive ALS in Brazil. Parental consanguinity may thus be best regarded as a modifier of testing priority when accompanied by red-flag features (juvenile onset, sibling recurrence, UMN-predominant phenotype, or HSP/neuropathy/ataxia overlap), rather than as a standalone indication for broad sequencing in otherwise classical late-onset ALS.

Several limitations of this study should be acknowledged. The most important is the pragmatic, clinically directed nature of genetic testing, which introduced a substantial selection bias that we have characterized quantitatively (Table 1) but cannot fully resolve. Related to this, the absence of an unaffected control group means that *ATXN2* intermediate-length repeat frequencies are reported as observed proportions in this clinical cohort rather than as risk-factor estimates; comparison with population frequencies in admixed Brazilian controls would strengthen the attribution of risk. Comparisons across genetic groups (Figure 4) and across Byrne family history categories (Figure 3) are likewise descriptive, and the small subgroup sizes preclude formal inference of effect sizes. Ancestry was not directly genotyped, and our inferences regarding European/Iberian contributions to the *SOD1* variant spectrum should be verified by genome-wide ancestry analysis in patients carrying p.(Val120Leu) and p.(Asp91Ala). Finally, the retrospective design constrained the depth and standardization of clinical documentation across the cohort.

Despite these limitations, the present cohort captures the broader real-world clinical landscape of motor neuron disease in Brazil and complements protocol-based studies that, by design, characterize preselected populations under controlled conditions. Building on the gaps identified, prospective Brazilian cohorts with systematic NGS applied uniformly to all enrolled patients, genome-wide ancestry genotyping in patients carrying recurrent *SOD1* variants of potential European origin, and broader use of recessive-gene panels in patients from consanguineous backgrounds would meaningfully advance the field. Equally important is the sustained effort to enhance awareness among Brazilian neurologists, in both public and private practice settings, of the value of systematic genetic testing for diagnostic precision, family counseling, and equitable access to gene-targeted therapy.

## 5 Conclusion

In this 18-year multiregional Brazilian cohort of 1,911 patients followed across six SARAH Network rehabilitation hospitals, the diagnostic yield of comprehensive panel or exome testing in non-ALS8 patients was 19.7% (13 of 66), including roughly 5% of patients without recognized family history. Family history remained the dominant criterion driving testing referrals, but educational attainment also differed significantly between genetically tested and untested patients, pointing to socioeconomic, not only clinical, determinants of testing equity in Brazil. Independent replication of the 6.5% *ATXN2* intermediate-length (≥26 CAG) frequency reported by Tavares de Andrade et al. (19) in a multicenter Brazilian sample, and a *SOD1* variant spectrum compatible with European/Iberian contributions to admixed Brazilian ancestry, together define a Brazilian genetic profile that warrants confirmation by prospective studies. These observational findings reinforce the importance of systematic genetic testing for diagnostic precision, family counseling, and equitable access to gene-targeted therapy (7).

## Data Availability

The datasets generated and analyzed during this study contain individual-level clinical and genetic data from patients followed at the SARAH Network of Rehabilitation Hospitals. These data are not publicly available because public deposition was not covered by the ethics approval granted for this retrospective study (CAAE 09814119.9.1001.0022) and would be incompatible with Brazilian National Health Council Resolution 466/12, under which the requirement for written informed consent was waived on the condition that fully de-identified data be used solely for the purposes of the approved protocol. No data types subject to mandatory deposition (e.g., novel nucleotide sequences, genome assemblies, or structural data) were generated in this study; all reported variants are previously described and referenced to public databases where applicable. De-identified aggregate data supporting the findings are available from the corresponding author upon reasonable request and subject to approval by the SARAH Network Research Ethics Committee.
