# Supplementary material for: Genetic profile of motor neuron disease in a multiregional Brazilian cohort: an 18-year real-world experience in 1,911 patients
Source: Front Neurol. 2026 Aug 5;17:1883977. doi: 10.3389/fneur.2026.1883977 (PMC13487208; doi:10.3389/fneur.2026.1883977)
Supplement: Supplementary file 1 [file Table_1.docx]

Supplementary Material

**Supplementary Figure S1.** Genetic burden of the SARAH Network motor neuron disease cohort. Proportion of each genetic finding among genetically tested patients (n = 383) and among the full cohort (n = 1,911). Bars show paired denominators for each finding; absolute case counts (n) are indicated. *ATXN2* intermediate-length expansions are shown separately (hatched) as a risk factor rather than a pathogenic/likely pathogenic variant.

**Supplementary Table S1.** Composition of multigene panels used across the study period (n = 61 panel-tested patients).

| **Panel size (genes)** | **Patients, n (%)** | **Period of use** | **ANXA11 included** | **Laboratory** |
| --- | --- | --- | --- | --- |
| 18 | 43 (70.5) | 2022–2025 | No | Lab A |
| 33 | 3 (4.9) | 2021–2022 | No | Lab B |
| 43 | 5 (8.2) | 2023–2024 | No | Lab C |
| 81 | 3 (4.9) | 2024–2025 | No | Lab A |
| 86 | 5 (8.2) | 2017–2023 | No | Lab D |
| 124 | 1 (1.6) | 2024 | Yes | Lab B |
| 169 | 1 (1.6) | 2020 | Yes | Lab E |

Multigene panels were performed at five accredited Brazilian laboratories (Lab A–E). All panels covered the core ALS genes most frequently assessed (*VAPB, SOD1, FUS, TARDBP, VCP, TBK1, SETX, ALS2, SPG11, SIGMAR1, OPTN, PFN1, UBQLN2*). *ANXA11* was included in only 2 of the 61 panels (the 124- and 169-gene panels). *C9orf72* repeat expansions were not detectable by standard panels and were assessed separately by repeat-primed PCR. Six additional patients underwent whole-exome sequencing (one also panel-tested), bringing the total to 66 unique panel- and exome-tested patients.

**Supplementary Table S2.** Variants of uncertain significance (VUS) identified in ALS-associated genes.

| **Gene** | **Reference transcript** | **Exon** | **Variant cDNA (HGVS)** | **Variant protein (HGVS)** | **Mutation type** |
| --- | --- | --- | --- | --- | --- |
| ***ALS2**** | NM_020919.3 | 10 | c.2111G>A | p.(Arg704Gln) | Missense |
| ***ALS2*** | NM_020919.4 | 20 | c.3256G>A | p.(Glu1086Lys) | Missense |
| ***OPTN*** | NM_001008212.2 | 12 | c.1396G>T | p.(Ala466Ser) | Missense |
| ***PFN1*** | NM_005022.3 | 2 | c.220A>G | p.(Ile74Val) | Missense |
| ***SETX*** | NM_015046.7 | 3 | c.146A>G | p.(Lys49Arg) | Missense |
| ***SETX*** | NM_015046.7 | 10 | c.2641G>A | p.(Glu881Lys) | Missense |
| ***SETX*** | NM_015046.7 | 10 | c.3117A>G | p.(Ile1039Met) | Missense |
| ***SETX*** | NM_015046.7 | 12 | c.3824G>A | p.(Arg1275His) | Missense |
| ***SOD1*** | NM_000454.5 | 5 | c.464A>C | p.(Ter155SerextTer6) | Stop-loss |
| ***TBK1*** | NM_013254.4 | 14 | c.1603G>A | p.(Ala535Thr) | Missense |

*****Patient also carries the *SOD1* p.(Val120Leu) likely pathogenic variant reported in Table 2. All variants were classified as VUS under the ACMG–AMP criteria; most were supported by activation of PM2 (rare in population databases) alone or in combination with BP4 (benign in silico predictions). ACMG, American College of Medical Genetics and Genomics; AMP, Association for Molecular Pathology; HGVS, Human Genome Variation Society; *VUS*, variant of uncertain significance.

**Supplementary Table S3.** Sensitivity analyses of the *ATXN2* intermediate-length CAG repeat carrier frequency in the SARAH Network motor neuron disease cohort, applying four repeat-length thresholds reported in the literature.

| **Cutoff (CAG repeats)** | **Carriers (n)** | **Total tested (n)** | **Frequency (%)** |
| --- | --- | --- | --- |
| ≥26 | 15 | 230 | 6.5 |
| ≥27 | 12 | 230 | 5.2 |
| 29–33 | 11 | 230 | 4.8 |
| ≥31 | 7 | 230 | 3.0 |

The principal threshold (≥26 CAG) was adopted in alignment with the only previous Brazilian study of *ATXN2* in ALS (19). The additional thresholds are reported in international cohorts (30,32) and in the meta-analysis by Sproviero et al. (31). The denominator (n = 230) corresponds to all patients in the cohort who underwent *ATXN2* CAG repeat sizing. CAG, cytosine-adenine-guanine trinucleotide repeat; *ATXN2*, ataxin-2 gene (locus 12q24.12; reference transcript NM_002973.4).

**Supplementary Table S4.** Clinical and demographic profile of consanguineous patients (n = 38) compared with the non-consanguineous patients (n = 1,873) in the SARAH Network motor neuron disease cohort.

| **Variable** | **Consanguineous (n = 38)** | **Non-consanguineous (n = 1,873)** | ***p*-value** |
| --- | --- | --- | --- |
| Sex, n (%) |  |  | 1.000 |
| Male | 21 (55.3) | 1,014 (54.1) |  |
| Female | 17 (44.7) | 859 (45.9) |  |
| Age at onset, years |  |  | 0.117 |
| Median (IQR) | 50.8 (38–61.2) | 55.4 (46–64) |  |
| Range | 22–82 | 17–84 |  |
| Age at onset by category, n (%) |  |  | 0.027 |
| <25 years | 1 (2.6) | 18 (1.0) |  |
| 25–39 years | 10 (26.3) | 240 (12.8) |  |
| ≥40 years | 27 (71.1) | 1,615 (86.2) |  |
| Phenotype, n (%) |  |  | 0.383 |
| Spinal-onset ALS | 24 (63.2) | 1,195 (63.8) |  |
| Bulbar-onset ALS | 5 (13.2) | 257 (13.7) |  |
| Cognitive-onset ALS | 0 (0.0) | 16 (0.9) |  |
| Respiratory-onset ALS | 0 (0.0) | 9 (0.5) |  |
| Primary lateral sclerosis | 1 (2.6) | 97 (5.2) |  |
| Progressive muscular atrophy | 7 (18.4) | 159 (8.5) |  |
| Flail arm syndrome | 0 (0.0) | 88 (4.7) |  |
| Flail leg syndrome | 1 (2.6) | 52 (2.8) |  |
| Family history (Byrne), n (%) |  |  | 0.002 |
| Definite | 1 (2.6) | 139 (7.4) |  |
| Probable | 0 (0.0) | 73 (3.9) |  |
| Possible | 4 (10.5) | 28 (1.5) |  |
| Sporadic | 33 (86.8) | 1,633 (87.2) |  |
| Region of birth, n (%) |  |  | 0.605 |
| Northeast | 18 (47.4) | 664 (35.5) |  |
| Southeast | 16 (42.1) | 1,032 (55.1) |  |
| Center-West | 2 (5.3) | 106 (5.7) |  |
| South | 1 (2.6) | 33 (1.8) |  |
| North | 1 (2.6) | 29 (1.5) |  |
| Outside Brazil | 0 (0.0) | 9 (0.5) |  |

*p*-values were calculated from Fisher's exact test (sex, phenotype, family history, region of birth), the chi-square test (age at onset by category), or the Mann–Whitney U test (age at onset, continuous). Bold p-values indicate significant differences (p < 0.05). ALS, amyotrophic lateral sclerosis; IQR, interquartile range.
